# Moiré cavity quantum electrodynamics

**DOI:** 10.1126/sciadv.adv8115

**Published:** 2025-05-21

**Authors:** Yu-Tong Wang, Qi-Hang Ye, Jun-Yong Yan, Yufei Qiao, Yu-Xin Liu, Yong-Zheng Ye, Chen Chen, Xiao-Tian Cheng, Chen-Hui Li, Zi-Jian Zhang, Cheng-Nian Huang, Yun Meng, Kai Zou, Wen-Kang Zhan, Chao Zhao, Xiaolong Hu, Clarence Augustine T. H. Tee, Wei E. I. Sha, Zhixiang Huang, Huiyun Liu, Chao-Yuan Jin, Lei Ying, Feng Liu

**Affiliations:** ^1^ State Key Laboratory of Extreme Photonics and Instrumentation, College of Information Science and Electronic Engineering, Zhejiang University, Hangzhou 310027, China.; ^2^ School of Physics, Zhejiang Key Laboratory of Micro-nano Quantum Chips and Quantum Control, Zhejiang University, Hangzhou 310027, China.; ^3^ International Joint Innovation Center, Zhejiang University, Haining 314400, China.; ^4^ School of Precision Instrument and Optoelectronic Engineering, Tianjin University, Tianjin 300072, China.; ^5^ Key Laboratory of Optoelectronic Information Science and Technology, Ministry of Education, Tianjin 300072, China.; ^6^ Laboratory of Solid State Optoelectronics Information Technology, Institute of Semiconductors, Chinese Academy of Sciences, Beijing 100083, China.; ^7^ College of Materials Science and Opto-Electronic Technology, University of Chinese Academy of Science, Beijing 101804, China.; ^8^ College of Physics and Electrical Information Engineering, Zhejiang Normal University, Hangzhou 310058, China.; ^9^ Key Laboratory of Intelligent Computing and Signal Processing, Ministry of Education, Anhui University, Hefei 230039, China.; ^10^ Department of Electronic and Electrical Engineering, University College London, London WC1E 7JE, UK.; ^11^ ZJU-Hangzhou Global Scientific and Technological Innovation Center, Zhejiang University, Hangzhou, Zhejiang 311200, China.

## Abstract

Quantum emitters are a key component in photonic quantum technologies. Enhancing single-photon emission by engineering their photonic environment is essential for improving overall efficiency in quantum information processing. However, this enhancement is often limited by the need for ultraprecise emitter placement within conventional photonic cavities. Inspired by the fascinating physics of moiré pattern, we propose a multilayer moiré photonic crystal with a robust isolated flatband. Theoretical analysis reveals that, with nearly infinite photonic density of states, the moiré cavity simultaneously has a high Purcell factor and large tolerance over the emitter’s position, breaking the constraints of conventional cavities. We then experimentally demonstrate various cavity quantum electrodynamic phenomena with a quantum dot in moiré cavity. A large tuning range (up to 40-fold) of quantum dot’s radiative lifetime is achieved through strong Purcell enhancement and inhibition effects. Our findings open the door for moiré flatband cavity–enhanced quantum light sources and quantum nodes for the quantum internet.

## Teaser

Tunable cavity quantum electrodynamics are enabled by moiré flatband photonic crystals.

## INTRODUCTION

Controlling individual single photons, i.e., the fundamental units of light described by Fock or number states (*1*), generated from a quantum emitter (*2*–*4*), is one of the major challenges in a wide range of disciplines from quantum optics (*5*) to quantum information technologies (*6*). An efficient approach to manipulating single-photon emission rates and wave packets is by artificially modifying photonic environments surrounding quantum emitters, since the emission properties are dictated by these photonic modes. Given their ability to reshape the spatial and frequency distribution of electromagnetic waves, optical cavities stand out as the most powerful and versatile tool for coherent single-photon control, forming the field of cavity quantum electrodynamics (cavity-QED) (*7*).

Traditionally made of general mirrors, cavities confine light waves at various scales. At macroscale, Fabry-Pérot cavities use traditional mirrors to trap light, while at the mesoscopic scale, cavities in nanophotonics use defects in photonic crystals (PhCs) or distributed Bragg reflectors (DBRs) for realizing similar confinement. In the latter case, traditional mirrors are replaced with effective optical “walls” such as the PhCs with a frequency bandgap or DBRs, in both of which electromagnetic fields exponentially decay beyond the cavity boundary. These approaches have found success in various areas. In recent years, cavity-QED has delved into the intricate interplay between quantum emitters and fine-designed optical cavities, revealing a range of phenomena such as the Purcell effect in the weak coupling regime (*8*–*10*), strong coupling (*11*–*13*), and dipole-induced transparency (*14*–*16*). In addition, more exotic cavities with specialized functions are proposed to enhance the photon emission such as photonic hyperbolic metamaterials (*17*) and surface plasmon in metallic structures (*18*) or to slow down the photon emission using photonic structures with unique photonic dispersion relationships such as the specific PhCs with Dirac (*19*) or Weyl (*20*, *21*) dispersion relationships and near-zero index materials (*22*). In general, the Purcell effect predicts that, overall, the photon emission rate of a system is inversely proportional to the mode volume while directly proportional to the *Q* factor (*23*).

This implies that to sustain a high spontaneous emission rate from a quantum emitter, a small mode volume and a large *Q* factor are essential. While a large *Q* factor is often constrained by fabrication imperfections and the fundamental diffraction limit for many cavity designs (*24*), a small mode volume presents additional challenges related to extremely precise emitter positioning to maximize its exposure to the local field (*25*).

Inspired by the fascinating physics of moiré superlattices in electronic and excitonic systems (*26*–*28*), its photonic counterpart (*29*–*35*) offers the potential for confining photons due to its isolated flatband dispersion relation. This theoretically leads to an infinite photonic density of states (DOS) at a fixed frequency, enabling simultaneous realization of an infinite *Q* factor and a large tolerance of emitter’s location within the cavity.

In this work, we propose using the moiré flatband photonics to modify the Purcell effect and experimentally manipulate single-photon emission from a semiconductor quantum dot (QD) within a robust quasi–one-dimensional (1D) triple-layer moiré cavity, eliminating the need for conventional mirrors and boundaries. Theoretical analysis shows that, because of its nearly infinite photonic DOS, both high Purcell factor and large tolerance over the emitter’s location can be obtained simultaneously. The formation of the flat photonic band and resulting light localization are confirmed by the photoluminescence (PL) spectra and mapping. A large tuning range (from 42 ± 1 to 1692 ± 7 ps) of the QD’s radiative lifetime is achieved while scanning the detuning between the QD and the moiré cavity, with an experimentally realized *Q* factor of 3523. The QD and moiré PhC fabricated from III-V semiconductor is grown directly on silicon. Our work demonstrates cavity-QED with moiré PhC, opening the door toward moiré flatband cavity–enhanced quantum optical devices compatible with silicon photonic platform (*36*–*39*).

## RESULTS

### Quantum emitter in flatband photonics

**Fig. 1.**
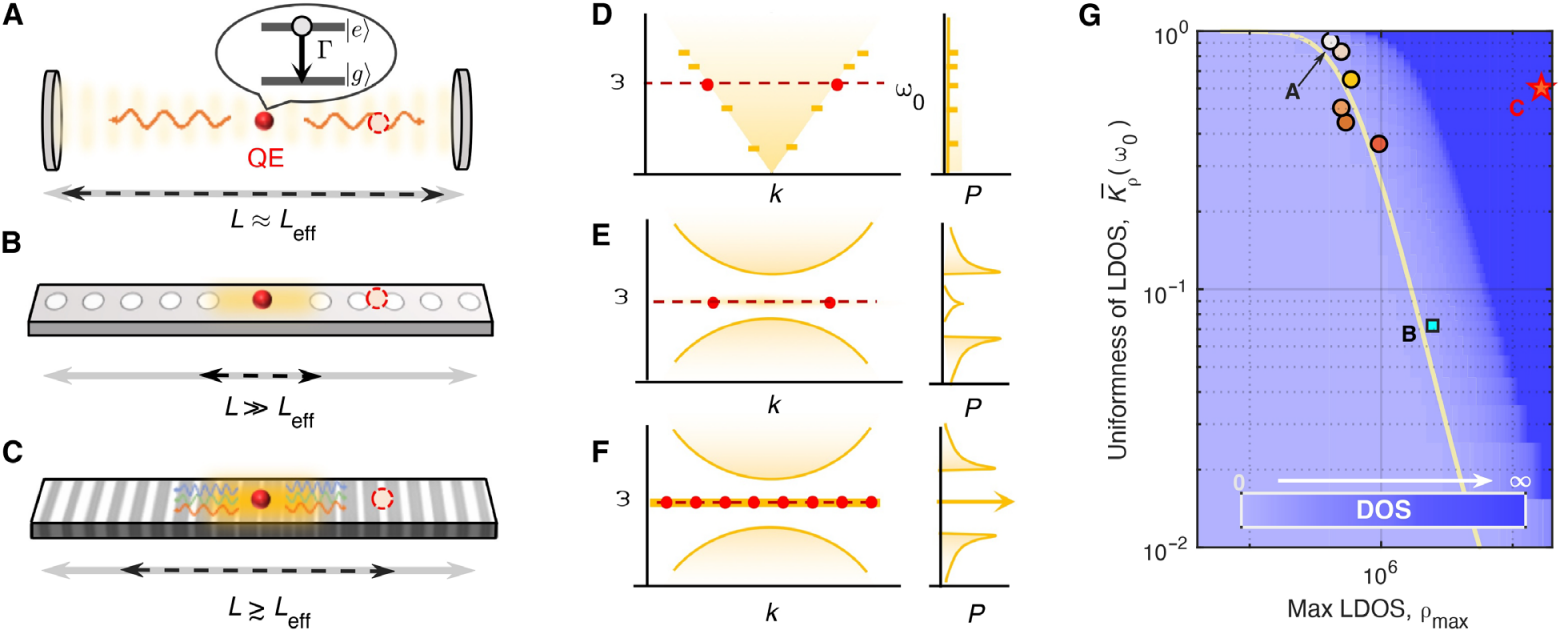
Schematics of photon emission in various photonic structures. A quantum emitter (QE) marked by a red filled circle respectively placed in (**A**) a traditional Fabry-Pérot cavity, (**B**) a 1D defect PhC cavity, and (**C**) a moiré PhC cavity. Red dashed circles stand for quantum emitters positioned at nonoptimal sites. Black dashed double arrows represent the effective length of cavities with strong LDOS, while gray double arrows denote the full lengths of photonic structures. A quantum emitter is a quantum system with two energy levels: a ground state and an excited state, as illustrated in the inset of (A). When the quantum system transitions from the excited state to the ground state, it emits a single photon with a spontaneous emission rate Γ. Left and right panels of (**D**) to (**F**) represent the dispersion relations and DOS P(ω) of photonic structures in (A) to (C), respectively. Red dots denote the effective modes in (A) to (C), and red dashed lines mark the transition frequency of the quantum emitter $\omega_{0}$. In the right panel of (E), the DOS inside the defect PhC cavity and in the bandgap PhC regime are denoted by solid yellow curves. (**G**) Schematically shows the uniformity of LDOS [${\bar{K}}_{\rho }=3-\text{Kurt}\rho \left(\omega_{0},x\right)$] versus the maximum value of LDOS [$\rho_{m}\left(\omega_{0}\right)$] for different fixed DOSs. Circles and squares represent the numerical results of the L3, L5, L7, L10, L15, and L20 and H1 defect PhC cavities, respectively, from top to bottom. Here, we use the L20 cavity as an analogy to the traditional Fabry-Pérot cavity in (A). The orange star represents the moiré PhC cavity. See numerical details in the Supplementary Materials.

Here, we focus on quasi-1D systems as shown in Fig. 1 (A to C), with their corresponding dispersion relations and DOS P(ω) are shown in Fig. 1 (D to F). We suppose that the photon volume is V≈AL, where A is the average cross-sectional area of quasi-1D structure and L is the length of the photonic structure or the period of PhC. The spatial confinement of a single photon by generic “mirrors” is radically determined by the photonic DOS P(ω) of the photonic structure, as the spontaneous emission rate of a quantum emitter Γ is proportional to the local DOS (LDOS). The photonic DOS and LDOS of a quasi-1D structure are respectively given by (see details in the Supplementary Materials)

$$P\left(\omega \right)=\int_{0}^{L}\rho \left(\omega ,x\right)\mathrm{dx}$$ (1)

and

$$\rho \left(\omega_{0},x\right)\approx \frac{\hbar }{4\mathrm{\pi A}}\sum_{n}\int_{\left\{k:\omega_{k}=\omega_{0}\right\}}\frac{{|\epsilon_{n,k}\left(x\right)|}^{2}}{v_{g}\left(k\right)}\mathrm{dk}$$ (2)

where $v_{g}\left(k\right)$ is the group velocity and $\epsilon_{n,k}\left(x\right)$ is the electric field density of the eigenmode (n,k). Here, k denotes the momentum, n represents the photonic band index in a PhC, and x represents the location of the quantum emitter. In general, the uniformity of LDOS over the spatial dimensions and the maximum LDOS are two important properties. The latter one is denoted by $\rho_{m}$. The former one, depicted by the uniformity ${\bar{K}}_{\rho }\equiv 3-\text{Kurt}\rho \left(\omega_{0},x\right)$, indicates the tolerance for quantum emitter placement in a photonic structure exhibiting a strong spontaneous emission rate. Here, Kurt(⋅) represents the normalized kurtosis function.

On the basis of the relationship in Eqs. 1 and 2, we find that for a fixed LDOS at the quantum emitter’s transition frequency $\omega_{0}$, there exists a trade-off between the spatial uniformity of the LDOS and the maximum LDOS $\rho_{m}$ within a general quasi-1D photonic structure, as illustrated by the contour diagram in Fig. 1G. This agrees with the empirical conclusion that a defect PhC cavity with a small effective mode volume (${\mathrm{AL}}_{\text{eff}}$) has a stronger enhancement of the spontaneous emission rate for a quantum emitter (or LDOS). For instance, the defect PhC cavities in Fig. 1 (B and E) exhibit a finite LDOS at the resonant frequency $\omega_{0}$. The local field can be moderately enhanced by decreasing the number of filling holes from multiple holes (L3, L5, L7, L10, L15, and L20) to one hole (H1). This enhancement is accompanied by a decrease in the effective photon volume and results in a reduced ${\bar{K}}_{\rho }$. The theoretical prediction can be confirmed by our numerical simulations, as shown in Fig. 1G. A similar trend can be observed in Fabry-Pérot cavities (see Fig. 1, A and D).

Now, we investigate an idealized scenario: a PhC structure with an isolated flatband dispersion relation, as shown in Fig. 1 (C and F). This configuration yields a divergent DOS in the frequency domain and the localization in real space (*40*), as illustrated in Fig. 1 (F and C), respectively. This implies that the spontaneous emission rate can reach an exceptionally high value when the quantum emitter is placed in suitable locations, while the uniformity of LDOS can be maintained at a reasonable level or, in other words, the high LDOS and large mode volume can be achieved simultaneously. Then, we numerically confirm an optimally designed moiré PhC structure described in the following text. As shown in Fig. 1G, this structure can exhibit a $\rho_{m}$ that is nearly two orders of magnitude higher than that of the L20 cavity, while maintaining the same level of ${\bar{K}}_{\rho }$ to the L20 cavity (see details in the Supplementary Materials).

Here, we emphasize that if the quantum emitter is located in a nonoptimal regime, i.e., its LDOS $\rho \left(x_{0}\right)$ is not at the maximum LDOS position (as illustrated by the dashed circles in Fig. 1, A to C), then the behavior changes. For a Fabry-Pérot cavity, the spontaneous emission rate is slightly modified by changing the quantum emitter’s position. In the case of a defect cavity, if the quantum emitter is placed out of the defect, then the emission is substantially suppressed. However, for an ideal isolated flatband, the emission rate of a quantum emitter can be large at most locations due to its infinite DOS at a fixed frequency $\omega_{0}$. On the other hand, in the frequency domain, the flatband PhC exhibits maximum DOS, enabling it to function as a quantum emitter switch controlling both ultrafast and ultraslow photon emission. Although the moiré structure exhibits much higher positional tolerance compared to defect cavities, its unique characteristics result in relatively weak electric field intensity or LDOS near the AB nodes—where the inner and outer holes are most offset. Consequently, the enhancement of the radiative rate in these regions is minimal.

### Moiré flatband cavity

**Fig. 2.**
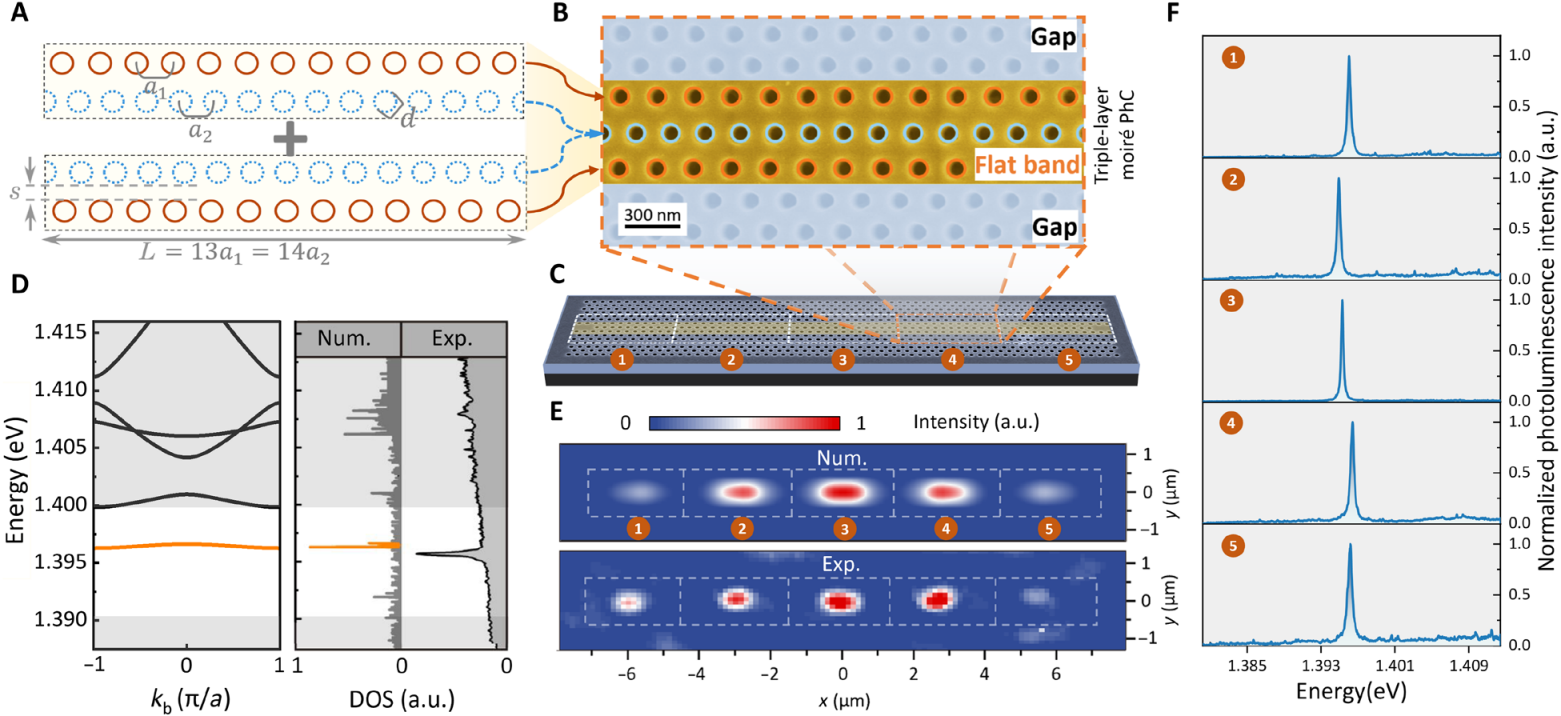
Design and characterization of moiré flatband cavity. (**A**) Two unit cells (gray dashed rectangles) of 1D moiré PhC composed of two 1D PhCs (brown and blue circles) with slightly different lattice constants ($a_{1}=209.1$ nm, $a_{2}=194.1$ nm). Other structural parameters: *d* = 133 nm, *s* = 95 nm, and *L* = 2718 nm. (**B**) SEM image of a fabricated triple-layer moiré PhC unit cell formed by combining two unit cells shown in (A). (**C**) SEM image of a moiré PhC consisting of five unit cells (white dashed rectangles). (**D**) Right: Comparison of calculated and experimentally measured photon DOS. The latter is obtained by spatially integrating PL spectra within a moiré PhC unit cell. The orange color indicates the flatband mode. a.u., arbitrary units. (**E**) Field spatial distribution of moiré flatband modes. Top: Numerical calculation accounting for the spatial resolution of the subsequent optical measurement. Bottom: PL map acquired by scanning the excitation and collection spots over the moiré PhC and recording the maximal PL intensity within 1.3939 to 1.3978 eV. The full width at half maximum (FWHM) of the excitation/collection spot is ~1.5 μm. (**F**) PL spectra of moiré cavity mode measured at centers of five moiré PhC unit cells marked in (C) under high-power above-barrier excitation. All experiments in this study are performed at *T* = 3.6 K. The *Q* factor of moiré cavity modes 1 to 5 are 3309, 3412, 5026, 3134, and 2602, respectively.

To study the single-photon emission of a QD embedded in a flatband moiré PhC, first, we design and fabricate a quasi-1D moiré PhC structure. This is composed of two types of 1D PhCs depicted by two lines of blue and brown circles in Fig. 2A. The lattice constants ($a_{1}$ and $a_{2}$) of the two 1D PhCs satisfy the condition $L=13a_{1}=14a_{2}$, which is a key requirement for the formation of a moiré PhC. The separation between two 1D PhCs, referred to as the magic distance (*s*), determines the flatness and frequency of the resulting moiré flatband. This moiré PhC confines light waves along the axis of the 1D PhC. We further introduce a triple-layer moiré PhC design (see Fig. 2B) by combining the two aforementioned structures. Such a multilayer moiré PhC is more robust to lattice constant variations, preserving a higher *Q* factor (see fig. S6) (*41*).

In addition, to achieve in-plane 2D confinement, we expand 1D PhCs on both top and bottom sides (shaded areas in Fig. 2B), providing light confinement in the longitudinal direction. Numerical simulations indicate a quality factor $Q=2.16\times 10^{4}$ (see details in the Supplementary Materials). Figure 2C shows the scanning electron microscopy (SEM) image of the moiré PhC consisting of five unit cells (labeled “1” to “5”) fabricated within a suspended gallium arsenide (GaAs) membrane containing indium gallium arsenide (InGaAs) QDs.

The entire device, including the QD and moiré PhC, is fabricated from III-V semiconductor grown directly on a silicon substrate using the molecular beam epitaxy technique (see details in the Supplementary Materials). This heterogeneous integration approach is technically demanding because of the difficulty of growing high-quality crystals on a lattice-mismatched substrate. However, it is a key step toward large-scale integrated quantum photonic circuits based on mature silicon photonic platform (*36*–*39*), which are currently limited by the absence of high-performance deterministic quantum light sources due to the indirect bandgap of silicon. Although the current QD emission wavelength lies above the silicon bandgap, it can be shifted to telecom bands via compositional tuning, strain engineering, and size control, as demonstrated in silicon-based epitaxial growth (*42*, *43*), ensuring compatibility with the silicon photonic platform.

To verify the dispersion relation of the designed moiré PhC, we perform the full-wave simulation, yielding a nearly flatband across the entire momentum space (see the orange curve in the left panel of Fig. 2D). The existence of such a nearly flatband is further confirmed by the consistency of the calculated photonic DOS as shown in Fig. 2D (Num.) and the peak of the spatially integrated PL spectrum of a moiré PhC unit cell measured under high-excitation laser power (see Fig. 2D, Exp.). All measurements in this work were conducted at *T* = 3.6 K.

One of the most interesting consequences of the flatband is the localization of light. This phenomenon is demonstrated and cross-checked by the spatial field distribution and PL spectra. The calculated field distribution shows that each unit cell of the moiré PhC acts as a cavity. The light field is well confined within five unit cells (see Fig. 2E, Num.). This simulation accounting for the spatial resolution (1.5 μm) of our optical measurement agrees well with the PL map measured by scanning the overlapping excitation and collection spots across the fabricated moiré PhC (see Fig. 2E, Exp.). In addition, the spectrally resolved PL signal acquired at the center of each moiré PhC unit cell exhibits a distinct peak with a maximum *Q* factor of 5026 at the energy of around 1.396 eV (see Fig. 2F). Again, this confirms the light localization in the moiré PhC. The minor variations in resonant frequencies of moiré cavities are attributed to slight differences in lattice constants of each PhC unit cell caused by nanofabrication imperfections, while the variations in *Q* factors for different cells are affected by the boundary condition (see the simulation result in Fig. 2E) and the fabrication error (see fig. S7) (*44*). The sufficient uniformity of moiré cavity modes demonstrates their high potential for constructing scalable arrays of identical cavity-enhanced quantum light sources.

### Control of single-photon emission

**Fig. 3.**
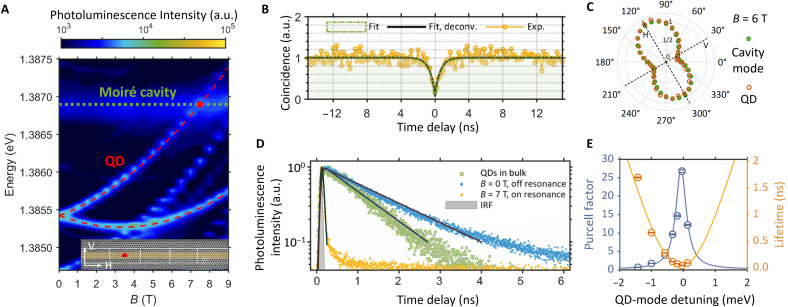
Manipulation of single-photon emission from a QD in moiré flatband cavity. (**A**) Magnetic field–dependent PL spectra of a QD and moiré cavity mode. The QD emission is split into two branches in an external magnetic field applied parallel to the QD growth axis (Faraday geometry). The higher-energy branch is tuned to be resonant with the moiré cavity mode at *B* = 7 T. The inset depicts a moiré cavity composed of five superlattice periods, with a trapezoid dot marking the QD position. White arrows indicate the horizontal (H) and vertical (V) polarization directions. (**B**) Second-order correlation measurement of single-photon emission from the QD under p-shell excitation. The black curve is obtained after deconvolving the detection response function from the green fit, yielding a single-photon purity of 0.93 ± 0.09. The uncertainties correspond to one SD from the fit. (**C**) Polarization of the emission from the QD (brown) and moiré cavity mode (green) characterized at *B* = 6 T. The polarization of both the QD and moiré cavity mode are dominantly along the longitudinal direction denoted as H in Fig. 1A (inset). (**D**) Time-resolved PL (TRPL) of the QD measured using an SNSPD. Gray, instrument response function (IRF) with an FWHM of 71 ± 1 ps; blue (orange), single QD detuned (resonant) with moiré cavity mode under LA phonon–assisted excitation; green, QD ensemble in bulk under above-bandgap excitation; black curves, single exponential fit. (**E**) QD-cavity detuning dependence of Purcell factor and QD lifetime. Solid lines, Lorentzian fit with a fixed FWHM. Error bars represent the uncertainty extracted from exponential fitting.

Following the characterization of the moiré PhC, we proceed to manipulate the spontaneous emission of a quantum emitter using the moiré flatband cavity. The first step is to identify a QD coupled with a moiré cavity. Figure 3A shows the magneto-PL spectra of a QD located in a moiré cavity. The position of the QD is indicated by the red trapezoid in the insert. The presence of both the QD emission (indicated by red dashed lines) and the moiré cavity mode (indicated by the green dotted line) in the same spectra confirms the spatial overlap between them. To clearly distinguish QD emission from the cavity mode, we use a very-low-excitation laser power. Under this condition, the PL intensity does not exhibit significant enhancement at cavity resonance. This behavior is attributed to (i) an excitation rate much lower than the Purcell-enhanced QD spontaneous emission rate and (ii) dominant QD emission into in-plane slab cavity modes at resonance, reducing collection efficiency in our top-side measurement configuration (*45*). The single-photon nature of the QD emission is verified in a standard Hanbury Brown and Twiss setup (*46*). Figure 3B presents a typical result showing strong antibunching and a single-photon purity of 0.93 ± 0.09 without any background subtraction, which unambiguously proves that the PL signal originates from a quantum emitter.

Moreover, the coupling between the QD and moiré cavity can be proved by the polarization measurement. The polarization-dependent PL intensity is measured by rotating the half-wave plate angle in front of a linear polarizer (see details in the Supplementary Materials). Typically, because of the Zeeman effect, the emission of an In(Ga)As QD subjected to a strong magnetic field in Faraday geometry splits into two branches with opposite circular polarizations (*47*). In contrast, modified by the moiré cavity, here, the photon emission from the upper branch in Fig. 3A exhibits predominantly linear polarization. In particular, its polarization measured at a high magnetic field of *B* = 6 T aligns well with that of the cavity mode, as shown in Fig. 3C. The polarization of both the QD and moiré cavity mode is mainly along the longitudinal axis of the cavity, denoted as H in Fig. 3A (inset). Therefore, this observation can be attributed to the QD–moiré cavity coupling, where the cavity mode dictates the polarization of the QD emission.

Last, we demonstrate the control over the spontaneous emission of a quantum emitter by the moiré cavity. Figure 3D shows the time-resolved PL (TRPL) of the QD measured at different QD-cavity detunings using a superconducting nanowire single-photon detector (SNSPD) (*48*). At *B* = 7 T where the QD and cavity are on resonance (see Fig. 3A), the TRPL (yellow dots in Fig. 3D) measured under longitudinal acoustic (LA) phonon–assisted excitation (*49*, *50*) yields a radiative lifetime $T_{1}$ as short as 42 ± 1 ps (50 ± 1 ps) with (without) deconvolving the instrument response function [full width at half maximum (FWHM) = 71 ± 1 ps]. This lifetime corresponds to a 27-fold (22-fold) emission rate enhancement compared with the average lifetime $T_{1}^{\prime }=1121\pm 3$ ps (green dots) for QD ensembles in GaAs bulk measured under above-barrier excitation. While at *B* = 0 T with the QD far detuned from the moiré cavity mode, $T_{1}$ slows down to 1692 ± 7 ps (blue dots in Fig. 3D) due to the Purcell inhibition (*51*–*54*).

Figure 3E summarizes the dependence of $T_{1}$ and the Purcell factor ($F_{p}=T_{1}^{\prime }/T_{1}$) on the QD-cavity detuning. The experimental data can be well fitted using the model describing the Purcell effect (*55*) with the measured cavity linewidth (0.394 meV). As shown in Fig. 3E, $T_{1}$ varies by more than one order of magnitude over a detuning range of 1.427 meV, demonstrating the effective control over QD’s spontaneous emission by the moiré cavity. In addition, Purcell enhancement is also observed with another QD coupled to a separate moiré cavity (see details in the Supplementary Materials).

## DISCUSSION

In summary, we have investigated cavity-QED with a moiré flatband PhC containing a quantum emitter. The flatband formation in moiré PhC can be understood as a result of the interference of multiple optical modes (*56*–*58*). Compared to conventional cavities, e.g., Fabry-Pérot cavity and PhC defect cavities (*23*), one of the key advantages of moiré flatband PhC is the extremely high photonic LDOS. This enables efficient control over the QD’s emission properties, including the polarization and radiative lifetime, as confirmed by the cavity-dominated polarization and a 40-fold tuning in radiative lifetime. This large tuning range is attributed to the pronounced Purcell enhancement and Purcell inhibition effects (*51*, *53*).

Photonic bound states in the continuum (BICs) were also proposed with ultralarge DOS (*59*–*61*). Compared with BICs, the flatband formed in the moiré PhC lies within a photonic bandgap, allowing the emission from a quantum emitter with finite linewidth to be coupled into the flatband mode (*60*, *62*). By contrast, in the case of BICs, the portion of quantum emitter’s emission that is not strictly resonant with the BIC mode can leak into radiative continuum modes, limiting the efficiency of Purcell enhancement.

As an outlook, combining the planar moiré PhC with various solid-state quantum emitters, including III-V QDs (*47*), color centers in diamond (*63*), 2D materials (*64*), and perovskite nanocrystals (*65*), could enable the development of arrays of cavity-enhanced on-chip quantum light sources (*2*, *55*, *66*–*69*), essential for large-scale quantum photonic circuits (*36*). The high Purcell factor of moiré cavities not only enhances photon emission rates but also improves photon indistinguishability by mitigating dephasing from phonons and charge noise. In addition, integrating the moiré PhC cavities with fast-light waveguides by optimizing the dispersion properties of both systems for efficient phase matching may provide an effective route for on-chip integration (*70*). Further improving the *Q* factor may achieve strong coupling between quantum emitters and flatband photonic structure (see numerical estimation in the Supplementary Materials), with potential applications including quantum gates (*71*, *72*), nondestructive photon detection (*73*), multiphoton graph states generation (*74*), ultrafast single-photon optical switch (*75*), and quantum nodes for quantum internet (*76*).

## MATERIALS AND METHODS

### Wafer structure and sample fabrication

The sample is fabricated on an InGaAs QD wafer grown by molecular beam epitaxy on a Si substrate. The wafer structure and detailed fabrication process are shown in fig. S1. The QDs are embedded at the center of a 140-nm GaAs membrane, with a sacrificial layer positioned underneath. After cleaning the wafer with acetone and isopropanol, an EBL (Electron Beam Lithography) resist (ARP-6200.13) is spin coated onto the surface. The moiré pattern is then defined in the resist using electron beam lithography (RAITH VOYAGER EBL system). Next, inductively coupled plasma etching (Oxford PlasmaPro 100 Cobra 180) is used to transfer the pattern into the GaAs layer. Last, wet etching is carried out to release the membrane, resulting in a suspended GaAs slab containing QDs.

### Optical measurement

The schematic of the setup for optical measurements is presented in fig. S1. The sample is located in a 3.6-K closed-loop cryostat (Attocube attoDRY), equipped with a magnetic field coil capable of generating up to *B* = 9 T of out-of-plane tunable magnetic field (Faraday geometry). For above-barrier excitation measurements, a 637-nm pump laser is used, produced by a continuous-wave (CW) diode laser (Thorlabs, LP637). For p-shell excitation, the laser wavelength is set to 880 nm, generated by a tunable CW Ti:sapphire laser (M Squared Solstis). Emission signals are collected using a custom-built confocal microscope with a 0.85–numerical aperture objective lens. The collected photons are directed either to a spectrometer (Princeton Instruments, HRS-750) with a grating of 1800 grooves/mm for spectral analysis or to an SNSPD for rapid single-photon detection. In TRPL measurements, the excitation pulse is provided by a tunable Ti:sapphire laser (Coherent Chameleon), emitting 150-fs pulses with an 80-MHz repetition rate. These pulses are then shaped to 8 ps using a home-made 4*f* pulse shaper. The pulses are tuned to the QD LA phonon–assisted excitation sideband to minimize state preparation time jitter (*49*, *50*). The emitted single photons are filtered by double bandpass filters before being sent to the SNSPD. For field spatial distribution measurements, the excitation and the collection spots are precisely aligned (spot size, ~1.5 μm). A 2D raster scan with a step size of ~80 nm is performed using the xy-piezo nanopositioners (attocube, ANPx101) below the sample.

## Supplementary Materials

### This PDF file includes:

Supplementary Text Figs. S1 to S8 References
