# Supplementary material for: Moiré cavity quantum electrodynamics
Source: Sci Adv. 2025 May 21;11(21):eadv8115. doi: 10.1126/sciadv.adv8115 (PMC12094201; doi:10.1126/sciadv.adv8115)
Supplement: Supplementary file 1 — Supplementary Text Figs. S1 to S8 References [file sciadv.adv8115_sm.pdf]

Science Advances
AAAS

Supplementary Materials for

Moiré cavity quantum electrodynamics

Yu-Tong Wang *et al.*

Corresponding author: Chao-Yuan Jin, jincy@zju.edu.cn; Lei Ying, leiying@zju.edu.cn; Feng Liu, feng_liu@zju.edu.cn

Sci. Adv. **11**, eadv8115 (2025)
DOI: 10.1126/sciadv.adv8115

This PDF file includes:

Supplementary Text
Figs. S1 to S8
References

1 Experimental setup

The experimental setup used in this work is shown in Supplementary Fig. S1. Measurements were conducted using a confocal microscope with the sample placed in a closed-cycle cryostat and excited by either picosecond pulses or cw lasers. The QD emission emission was collected via a single-mode fiber and directed to one of three parts: a spectrometer for the measurement of PL spectra, a TRPL setup for lifetime measurements, or an HBT setup for single-photon purity analysis.

For polarization-dependent measurements, we excite the QD with a weak above-barrier laser to ensure a clear distinction between the cavity mode and QD emission. We rotate the HWP in front of a linear polarizer in the collection optical path (see Fig. S1), which effectively varies the collection linear polarization basis. We then acquire a series of polarization-dependent emission spectra. By Gaussian fitting, we extract the integrated areas of the cavity and QD peaks as a function of the collection polarization angle, as shown in Fig. 3**C**.

2 Wafer structure and sample fabrication process

Figure S2 (a) illustrates the wafer structure of the InGaAs quantum dot sample. A single layer of InGaAs QDs is at the center of a 140-nm GaAs membrane. To create a suspended membrane, a 1-μm $Al_{0.6}Ga_{0.4}As$ sacrificial layer is grown to make the GaAs membrane suspended. Below this structure, short-period PhC (SPL) and strained layer PhC (SLS) layers facilitate the transition between the Si substrate and the III-V semiconductor.

The process for fabricating the flatband structure, depicted in Fig. S2 (b), involves the following steps: First, the pattern is defined using electron beam lithography with the photoresist ARP-6200.13, followed by development in ARP600-546 for 1 minute. Subsequently, inductively coupled plasma etching is performed with a BCl_3/N_2 ratio of 2:3 to transfer the pattern

into the GaAs layer, etching to a depth of 200 nm to ensure full penetration through the membrane. The sample is then immersed in a hydrofluoric acid solution (HF:DI =1:5) for 15 minutes to remove the sacrificial layer beneath the pattern, resulting in a suspended GaAs slab featuring a moiré flatband structure.

3 Theoretical derivation

3.1 Hamiltonians

We consider a quantum emitter (QE) embedding in PhC (PhC) structure. The QE can be modeled by a two-level system and its Hamiltonian is given by

$$\hat{H}_{\mathrm{QE}} = \omega_0 \hat{\sigma}^\dagger \hat{\sigma}, \tag{S1}$$

where ω_0 is the transition frequency of quantum dot and $\hat{\sigma}^\dagger$ ($\hat{\sigma}$) is the raising (lowering) operator. The Hamiltonian of PhC is written as

$$\hat{H}_{\mathrm{PhC}} = \sum_{n,\mathbf{k}} \omega_{n,\mathbf{k}} \hat{a}^\dagger_{n,\mathbf{k}} \hat{a}_{n,\mathbf{k}}, \tag{S2}$$

where $\omega_{n,\mathbf{k}}$ is the photon frequency for momentum $n, \mathbf{k}$. $\hat{a}^\dagger_{n,\mathbf{k}}(\hat{a}_{n,\mathbf{k}})$ is the creation(annihilation) operator. The light-matter interaction term is

$$\hat{H}_{\mathrm{int}} = \sum_{n,\mathbf{k}} \left[i g_{n,\mathbf{k}}(\mathbf{r}) \left(\hat{\sigma}^\dagger + \hat{\sigma} \right) \hat{a}^\dagger_k e^{in,\mathbf{k}\cdot\mathbf{r}} + \mathrm{h.c.} \right], \tag{S3}$$

where $g_{n,\mathbf{k}}(r) = \sqrt{\omega_{n,\mathbf{k}}/2\epsilon_0 V} \boldsymbol{\mu} \cdot \boldsymbol{\epsilon}_{n,\mathbf{k}}$ is the coupling between the photon labeled with $n, \mathbf{k}$ and the QE at position $\mathbf{r}$. Here, $\boldsymbol{\mu}$ is the dipole matrix element of the QE and $\boldsymbol{\epsilon}_{\mathbf{k}}$ is the electric field of the mode $\mathbf{k}$.

3.2 Spontaneous emission rate and LDOS

The spontaneous emission rate can be derived from perturbation theory, where the interaction is considered as the perturbation, thus the transition matrix element is given by

$$M_{\mathrm{FI}} = \langle \mathrm{F}| \hat{H}_{\mathrm{int}} |\mathrm{I}\rangle + \sum_{\alpha} \frac{\langle \mathrm{F}| \hat{H}_{\mathrm{int}} |\mathrm{R}_\alpha\rangle \langle \mathrm{R}_\alpha| \hat{H}_{\mathrm{int}} |\mathrm{I}\rangle}{E_{\mathrm{I}} - E_{\mathrm{R}_\alpha}} + \cdots . \tag{S4}$$

For small couplings, retaining terms up to the second term of expansion already achieves very high precision. The initial state and the final state are chosen to be the same, $|\mathrm{I}\rangle = |\mathrm{F}\rangle = |e; 0\rangle$. In the bracket '$e$' means the quantum dot is at the excited state, and the Arabic number indicates the number of photons in PhC. Two intermediate states are $|R_1\rangle = |g; 1_{n,\mathbf{k}}\rangle$ and $|R_2\rangle = |e; 1_{n,\mathbf{k}}\rangle$. The energy for state $|\mathrm{I}\rangle$, $|R_1\rangle$ and $|R_2\rangle$ are respectively $E_{\mathrm{I}} = E_{\mathrm{e}}$, $E_{\mathrm{R}_1} = \hbar\omega_{n,\mathbf{k}}$ and $E_{\mathrm{R}_2} = E_{\mathrm{e}} + E_{\mathrm{e}}^{(n)} + \hbar\omega_{n,\mathbf{k}}$. In our discussion, The energy of $|g; 0\rangle$ serves as the zero-point of energy. The final result is given by [20].

$$M_{\mathrm{FI}} = \sum_{n,\mathbf{k}} \left(g_{n,\mathbf{k}}(\mathbf{r}_m) g^*_{n,\mathbf{k}}(\mathbf{r}_n) \frac{1}{\omega_{n,\mathbf{k}} - \omega_0} + g^*_{n,\mathbf{k}}(\mathbf{r}_m) g_{n,\mathbf{k}}(\mathbf{r}_n) \frac{1}{\omega_{n,\mathbf{k}} + \omega_0} \right). \tag{S5}$$

Replace the sum of $\mathbf{k}$ by $V/(2\pi)^3 \int_{1\mathrm{BZ}} d^3\mathbf{k}$ and take the imaginary part, we obtain the spontaneous emission rate as

$$\Gamma(\omega_0) = \sum_n \int_{1\mathrm{BZ}} d^3\mathbf{k} \frac{\omega_{n,\mathbf{k}}}{16\pi^2\epsilon_0} |\boldsymbol{\mu} \cdot \boldsymbol{\epsilon}_{\mathbf{k}}|^2 \delta(\omega_{n,\mathbf{k}} - \omega_0) = \sum_n \frac{\omega_0}{16\pi^2\epsilon_0} \int_{\{\mathbf{k}:\omega_{n,\mathbf{k}}=\omega_0\}} \frac{|\boldsymbol{\mu} \cdot \boldsymbol{\epsilon}_{\mathbf{k}}|^2}{|v_g(\mathbf{k})|} dS_{\mathbf{k}}. \tag{S6}$$

For the case of quasi-1D PhC structure, only k_x direction has continuous dispersion relation. Thus, the integral over the iso-frequency surface is reduced to the integral over the momentum direction k along the x direction. Here, we use k to represent the momentum along the x direction. We assume that the electric field distribution at the $y - z$ cross section is uniform for each mode k. The cross-section area of the quasi-1D photonic structure is A. Then, the spontaneous emission can be re-written as

$$\Gamma(\omega_0) \approx \sum_n \frac{\omega_0}{4A\epsilon_0} \int_{\{k:\omega_k=\omega_0\}} \frac{|\boldsymbol{\mu} \cdot \boldsymbol{\epsilon}_{n,k}|^2}{v_g(k)} dk. \tag{S7}$$

In general, the relationship between the spontaneous emission rate and the photonic local density of state is given by

$$\Gamma(\boldsymbol{\mu} \to 1, \omega_0) = \sum_n \frac{\pi\omega_0}{\hbar\epsilon_0}\rho(\omega, \mathbf{r}), \tag{S8}$$

the LDOS is given by

$$\rho(\omega, \mathbf{r}) = \sum_n \int_{\omega_k=\omega_0} \frac{\hbar}{16\pi^2|v_g(\mathbf{k})|}|\boldsymbol{\epsilon}_{n,k}(\mathbf{r})|^2 dS_{\mathbf{k}}. \tag{S9}$$

For quasi-1D scenario, we have

$$\rho(\omega_0, x) \approx \frac{\hbar}{4\pi A}\sum_n \int_{k\in\{\omega_k=\omega_0\}} \frac{|\boldsymbol{\epsilon}_{n,k}(x)|^2}{v_g(k)} dk. \tag{S10}$$

3.3 Purcell factor

We use the general definition to derive the Purcell factor. At first, we consider the emission power

$$W = \frac{\omega}{2}\mathrm{Im}\left[\boldsymbol{\mu}\cdot\mathbf{E}(\mathbf{r}_\mathrm{s})\right], \tag{S11}$$

where $\boldsymbol{\mu}$ is the dipole element and $\mathbf{r}_s$ is its position. The electric field is given by the Helmholtz equation:

$$\nabla\times\nabla\times\mathbf{E}(\mathbf{r}) - \epsilon(\mathbf{r})k_0^2\mathbf{E}(\mathbf{r}) = \mathrm{i}\mu_0\omega\mathbf{j}(\mathbf{r}). \tag{S12}$$

Alternatively, we can express the electric field with the Green's function

$$\mathbf{E}(\mathbf{r}) = \mathrm{i}\mu_0\omega\int \mathbf{G}(\mathbf{r}, \mathbf{r}', \omega)\mathbf{j}(\mathbf{r}')\mathrm{d}\mathbf{r}', \tag{S13}$$

where the Green's function can be obtained from

$$\nabla\times\nabla\times\mathbf{G}(\mathbf{r}, \mathbf{r}', \omega) - \epsilon(\mathbf{r})k_0^2\mathbf{G}(\mathbf{r}, \mathbf{r}', \omega) = \mathbf{I}_{3\times3}\delta(\mathbf{r}-\mathbf{r}'). \tag{S14}$$

For a point-like quantum dipole, we have

$$W = \frac{\mu_0\omega^3}{2}|\mu|^2\mathrm{Im}\left[\hat{\mu}\cdot\mathbf{G}(\mathbf{r}_s, \mathbf{r}_s', \omega)\cdot\hat{\mu}\right]. \tag{S15}$$

In free space, the emission power is given by

$$W_0 = \frac{\omega^4}{12\pi\epsilon_0 c^3}|\mu|^2. \tag{S16}$$

Then, the Purcell factor is written as

$$F_P = \frac{W}{W_0} = \frac{6\pi}{k_0}\mathrm{Im}\left[\hat{\mu}\cdot\mathbf{G}(\mathbf{r}_s,\mathbf{r}'_s,\omega)\cdot\hat{\mu}\right]. \tag{S17}$$

With the eigenmodes $\mathbf{e}_n(\mathbf{r})$ of the Helmholtz equation, we can express the Green's function as

$$\mathbf{G}(\mathbf{r},\mathbf{r}',\omega) = c^2\sum_n \frac{\boldsymbol{\epsilon}_n(\mathbf{r})\otimes\boldsymbol{\epsilon}_n^*(\mathbf{r}')}{\omega_n^2-\omega^2-i\omega\gamma_n}. \tag{S18}$$

Here, the notation $\bigotimes$ denotes the dyadic product. γ_n is the damping rate of mode n. For a periodic structure, to make the mode more explicit, we rewrite the Green's function as

$$\mathbf{G}(\mathbf{r},\mathbf{r}',\omega) = \frac{Vc^2}{(2\pi)^3}\int\frac{\boldsymbol{\epsilon}_{n,\mathbf{k}}(\mathbf{r})\otimes\boldsymbol{\epsilon}_{n,\mathbf{k}}^*(\mathbf{r}')}{\omega_{n,\mathbf{k}}^2-\omega^2-i\omega\gamma_{n,\mathbf{k}}}d^3\mathbf{k}, \tag{S19}$$

where we use n to labelel the eigenenergy and $\mathbf{k}$ to label the Bloch vector in the 1st Brillouin zone. As ω is close to a flatband, the summation in Eq. (S19) becomes extremely large and thus it will lead to strong Purcell enhancement.

3.4 Coupling strength

The coupling strength between the flatband photonic mode and a quantum emitter can be calculated by

$$g(\mathbf{r}) = \sum_{\omega_{\mathbf{k}}\omega_0}\frac{\boldsymbol{\mu}\cdot\boldsymbol{\epsilon}_{\mathbf{k}}(\mathbf{r})}{\hbar} \tag{S20}$$

As the flatband mode for different wavevectors at the same frequency exhibits a similar $\mathbf{E}$-field distribution, we can approximate the coupling strength as

$$g(\mathbf{r}) \approx N_{\mathbf{k}}\frac{\boldsymbol{\mu}\cdot\boldsymbol{\epsilon}_{\mathbf{k}}(\mathbf{r})}{\hbar}, \tag{S21}$$

where $N_\mathbf{k}$ is the number of k points in the first Brillouin zone. For a finite-size structure in the experiment, this value is proportional to the least common multiple of the two lattice periods in the moiré structure and the total number of unit cells. For example, with a $31 : 32$ hole ratio in two columns and three unit cells, our numerical simulations predict a coupling strength g of approximately 24 GHz. This value is approaching the values achieved in the former works of L3 PhC cavities for strong coupling (*77–80*).

4 Numerical results for different PhC cavities

In Fig. S3, we present the distributions of the Purcell factor for the moiré structure, h1 cavity, and various L-type cavities. As shown, the moiré structure theoretically exhibits a significantly larger Purcell factor compared to conventional cavities, while still maintaining a considerable spatial extent. Theoretically, this feature breaks the conventional trade-off observed in traditional cavities, where the h1 and L3 cavities achieve relatively high Purcell factors but with small effective mode volume. Conversely, larger L-type cavities, such as L10, L15, and L20, offer a broader spatial extent while they have smaller Purcell factors. Same conclusions in LDOS distributions are shown in Fig. S4.

Figure S6(a) shows the bandgap area, which typically correlates with a high Q factor for the flatband within the bandgap. Figure S6(b) presents the numerical results for the Q factor of moiré cavity and L3 cavity. Considering material dissipation, we find that the Q factor of the moiré cavity varies very slowly with the hole diameter, similar to the behavior observed in the defect cavity. This suggests that fabricating the moiré structure may pose additional challenges compared to the defect cavity.

Figure S7 presents the robustness analysis for the traditiona moiré structure and the structure designed by us. Our design consists of three lines of holes with separations a_1, a_2, and a_1, respectively, while the traditional two-line design consists of two lines of holes with sepa-

rations a_1 and a_2. For a more practical simulation, we introduce an imaginary component to the refractive index of GaAs, which causes the Q factor to decrease by approximately three orders of magnitude. As the difference in hole separations $\Delta a = a_1 - a_2$ changes by a small amount (ś2.5%), our design shows a smaller and more linear change in the Q factor, while the traditional design exhibits a larger and non-linear change, indicating that the three-line design has lower sensitivity to the error of the Δa. We further evaluate the robustness of the structures against disorder in hole diameter d. As the errors in d increase, the Q factors of both structures decline. Notably, the Q factor of our structure decreases faster than that of the traditional structure, primarily due to the larger number of holes in our design, making it more susceptible to disorder in d. These results suggest that the Q factor being only in the thousands is mainly attributed to the fabrication errors in the hole diameter. By reducing the hole diameter error to a reasonable value, such as 4%, the Q factor can be significantly increased. Though challenging, such improvements in fabrication are still achievable.

Furthermore, we define a quantity, $A_{\mathrm{eff}}/A_{\mathrm{uc}}$, to quantify the enhancement tolerance to the QD position. Here, A_{eff} represents the effective area within a unit cell where the LDOS exceeds half the maximum LDOS of the L20 PhC defect cavity. A_{uc} denotes the area of a single unit cell. As shown in Fig. S5, the LDOS in moiré structures is remarkably higher than those in traditional cavities. Also, we have confirmed the average LDOS ρ_{eff} in the effective area A_{eff} for various cavities. Our analysis demonstrates that the moiré cavity exhibits a significantly enhanced average LDOS.

5 Purcell enhancement of QD B

To further validate the moiré cavity-enhanced QD fluorescence, we provide supplementary raw data from additional QD, labeled QD B, which is located in a moiré cavity adjacent to the one discussed in the main text, within the same chip. Figure S8(a) shows spectra including the cor-

responding moiré cavity mode with the fluorescence emission from QD B. The dominant peak observed in the QD emission spectrum is attributed to QD B. Utilizing above-barrier excitation, we measure a fluorescence lifetime of 141$\pm$3 ps (Fig. S8(b)), corresponding to a Purcell enhancement factor of approximately 8. Given the moiré cavity Q factor of 2191, precisely tuning QD B in resonance with the cavity mode would predict a Purcell factor around 13.8, as shown as the peak value in Fig. S8(c). We note that above-barrier excitation leads to long carrier relaxation time, typically hundreds of picoseconds (*81–84*), from higher-energy states to the lowest exciton state, obscuring the true Purcell factor (*55*). We anticipate that a reduced lifetime, hence a higher Purcell factor, can be measured if phonon-assisted or resonant excitation is employed, as demonstrated in the main text with QD A.

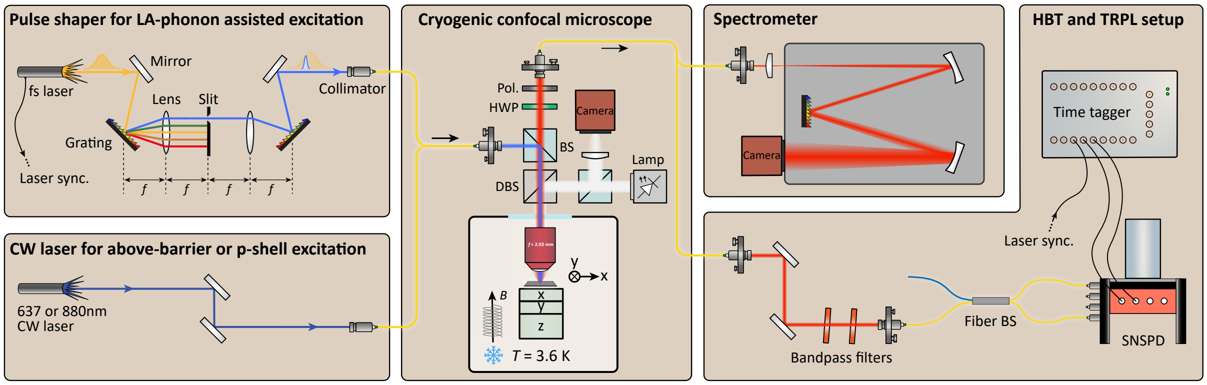

Figure S1: **Schematic of the setup for optical measurements.** Left panels: laser excitation part including $4f$ pulse shaping setup for LA-phonon-assisted excitation and CW lasers for above-barrier or p-shell excitation. Central panel: a home-built confocal microscope with the sample loaded in a closed-cycle cryostat (T = 3.6 K). Right panels: single-photon characterization part including spectrometer, HBT interferometer, and TRPL setup. Laser sync.: laser synchronization signal. Pol.: polarizer. HWP: half-wave plate. BS: beam splitter. DBS: dichroic beam splitter.

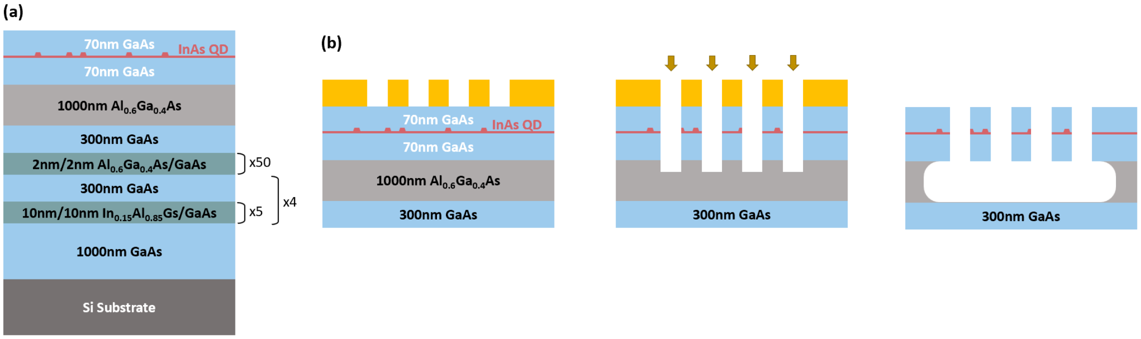

Figure S2: **Wafer structure and sample fabrication process.** (a) Wafer structure of the InGaAs quantum dot sample. (b) Process flow for patterning and etching to achieve a suspended GaAs slab.

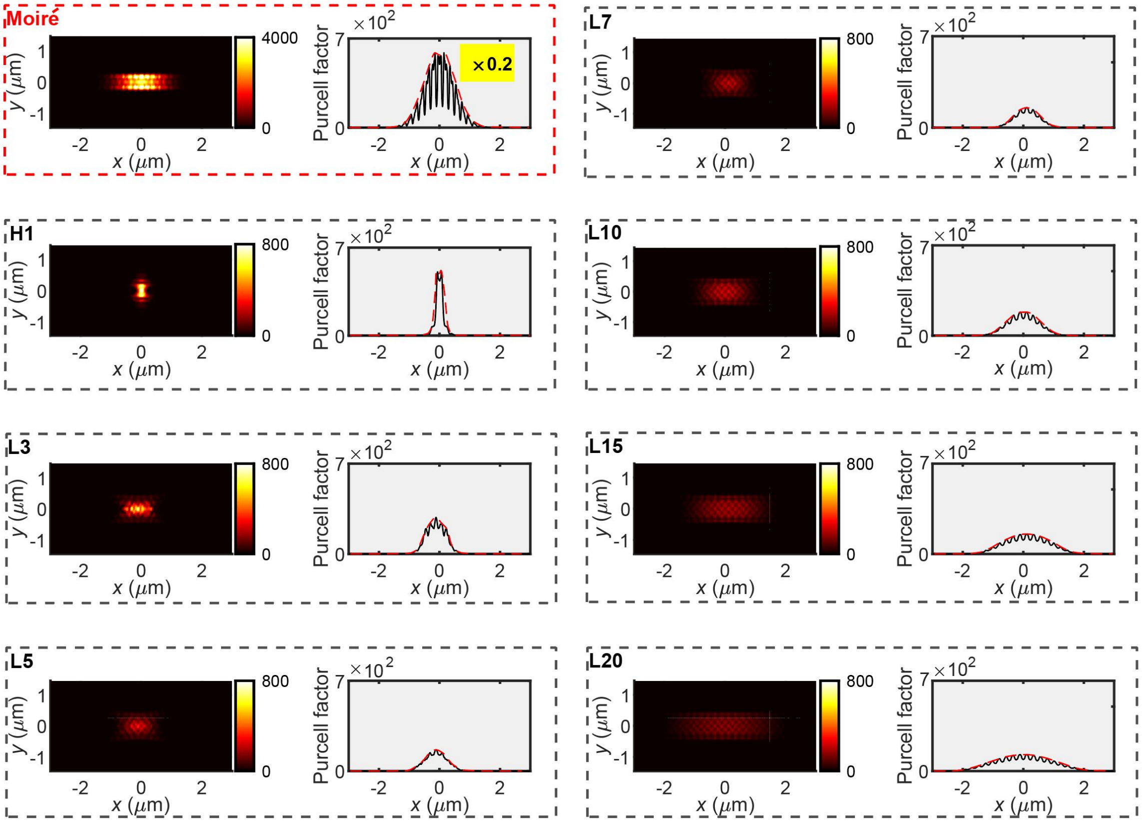

Figure S3: **Distribution of the Purcell factor for moiré lattice, comparing to conventional defect PhC cavities.** The left column presents the distribution in the x-y plane. The right column is the corresponding result after averaging over the range y = -400nm to 400nm, with the red dashed line representing the envelope of its distribution. For visualization purposes, the averaged Purcell factor for the moiré structure is scaled down by a factor of 5.

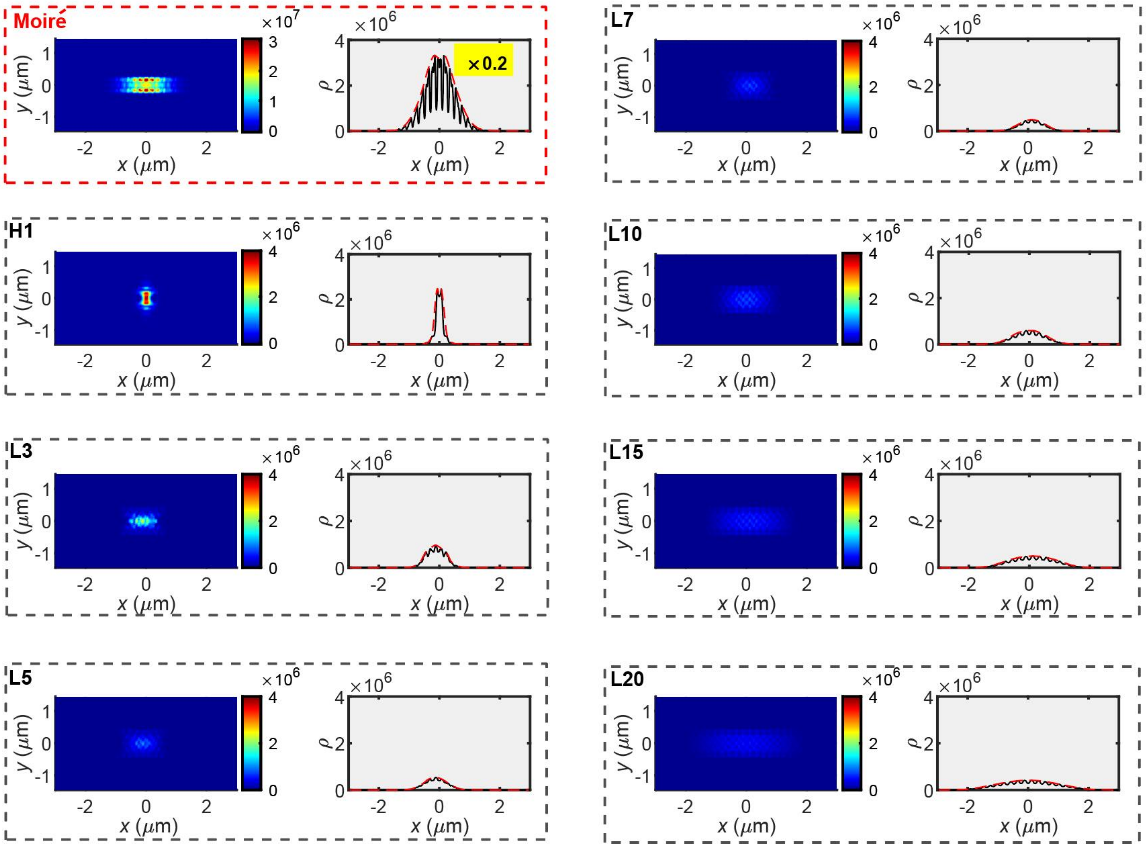

Figure S4: **Distribution of the LDOS for moiré lattice, comparing to conventional defect PhC cavities.** The left column presents the distribution in the x-y plane. The right column is the corresponding result after averaging over the range y = -400nm to 400nm, with the red dashed line representing the envelope of its distribution. For visualization purposes, the averaged LDOS values for the moiré structure are scaled down by a factor of 5.

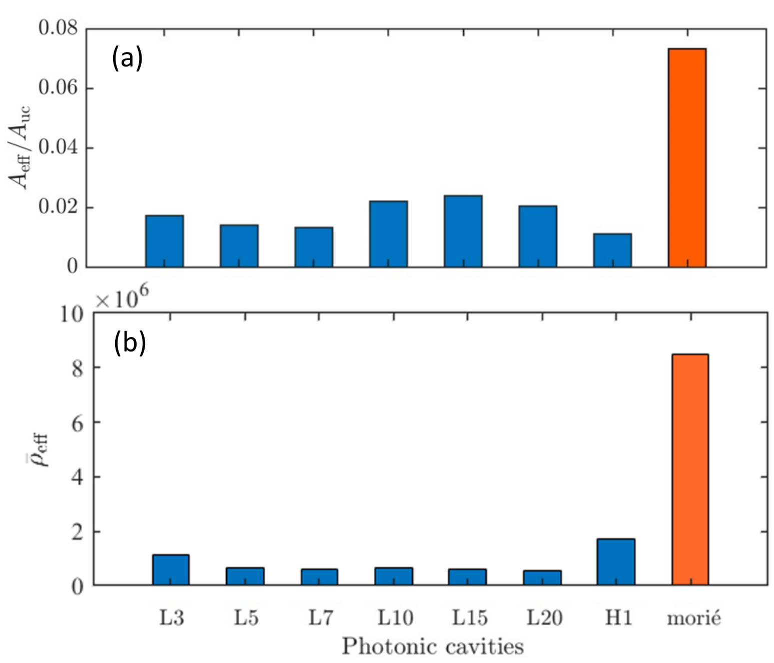

Figure S5: **Numerical histogram results for main properties of various photonic cavities.** (a) Effective area for Aeff over unit cell area. (b) Average LDOS on the effective area.

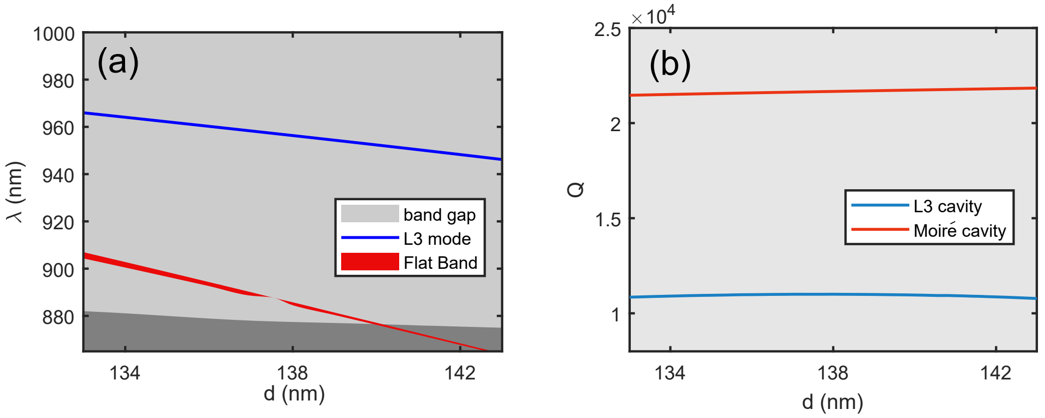

Figure S6: **Numerical results for varying hole diameter.** (a) Frequency shift of flatband and L3 mode as a function of hole diameter. The red band denotes the flatband mode and its thickness represents the bandwidth. The dark grey region represents the bulk modes while the light grey region stands for the bandgap. (b) Q factor versus hole diameter for the moiré and L3 cavities. The Q factor of both cavities shows minimal variation with changes in hole diameter.

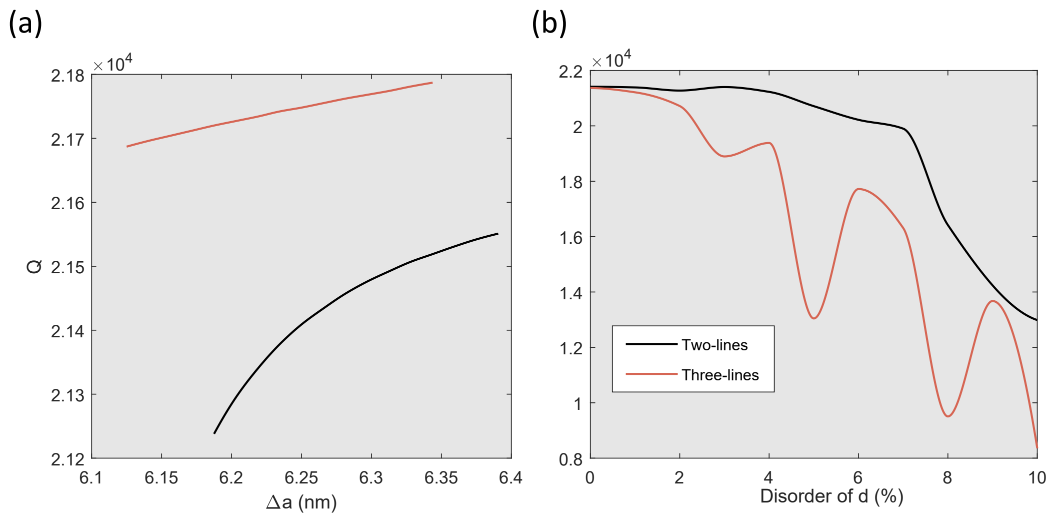

Figure S7: **Robustness test for two designs of moiré structures.** (a) The Q factors vs. the lattice constant difference Δa (b) The Q factors vs. Disorder of hole diameter d.

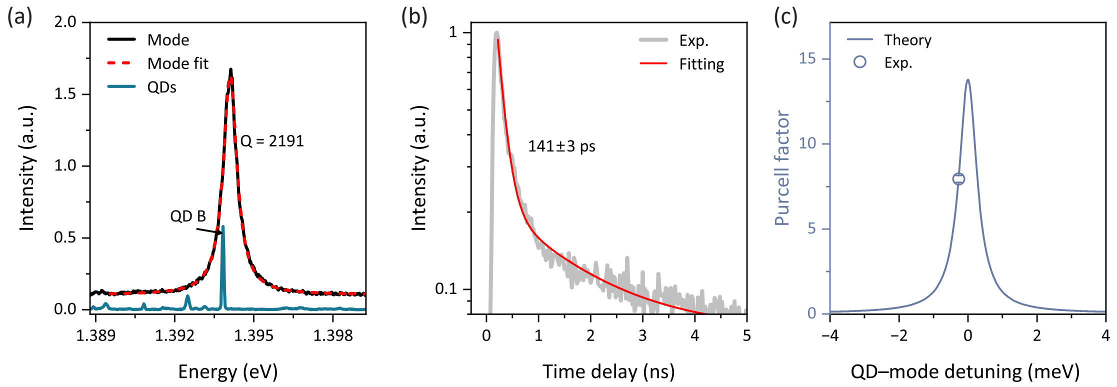

Figure S8: **Purcell enhancement of QD B.** (a) Spectra of QD B and another moiré cavity mode. (b) Time-resolved PL of the QD B (c), The Purcell enhancement factor of the QD B as a function of detuning.

REFERENCES AND NOTES

1. M. O. Scully, M. S. Zubairy, *Quantum Optics* (Cambridge Univ. Press) (2012).

2. N. Tomm, A. Javadi, N. O. Antoniadis, D. Najer, M. C. Löbl, A. R. Korsch, R. Schott, S. R. Valentin, A. D. Wieck, A. Ludwig, R. J. Warburton, A bright and fast source of coherent single photons. *Nat. Nanotechnol.* **16**, 399–403 (2021).

3. Y. O. Dudin, A. Kuzmich, Strongly interacting Rydberg excitations of a cold atomic gas. *Science* **336**, 887–889 (2012).

4. C. Kurtsiefer, S. Mayer, P. Zarda, H. Weinfurter, Stable solid-state source of single photons. *Phys. Rev. Lett.* **85**, 290–293 (2000).

5. I. A. Walmsley, Quantum optics: Science and technology in a new light. *Science* **348**, 525–530 (2015).

6. C. Couteau, S. Barz, T. Durt, T. Gerrits, J. Huwer, R. Prevedel, J. Rarity, A. Shields, G. Weihs, Applications of single photons to quantum communication and computing. *Nat. Rev. Phys.* **5**, 326–338 (2023).

7. H. Walther, B. T. H. Varcoe, B.-G. Englert, T. Becker, Cavity quantum electrodynamics. *Rep. Prog. Phys.* **69**, 1325–1382 (2006).

8. M. Arcari, I. Söllner, A. Javadi, S. Lindskov Hansen, S. Mahmoodian, J. Liu, H. Thyrrestrup, E. H. Lee, J. D. Song, S. Stobbe, P. Lodahl, Near-unity coupling efficiency of a quantum emitter to a photonic crystal waveguide. *Phys. Rev. Lett.* **113**, 093603 (2014).

9. P. Lodahl, S. Mahmoodian, S. Stobbe, Interfacing single photons and single quantum dots with photonic nanostructures. *Rev. Mod. Phys.* **87**, 347–400 (2015).

10. N. Somaschi, V. Giesz, L. De Santis, J. C. Loredo, M. P. Almeida, G. Hornecker, S. L. Portalupi, T. Grange, C. Antón, J. Demory, C. Gómez, I. Sagnes, N. D. Lanzillotti-Kimura, A. Lemaítre, A. Auffeves, A. G. White, L. Lanco, P. Senellart, Near-optimal single-photon sources in the solid state. *Nat. Photonics* **10**, 340–345 (2016).

11. R. Ohta, Y. Ota, M. Nomura, N. Kumagai, S. Ishida, S. Iwamoto, Y. Arakawa, Strong coupling between a photonic crystal nanobeam cavity and a single quantum dot. *Appl. Phys. Lett.* **98**, 173104 (2011).

12. J. P. Reithmaier, G. Sęk, A. Löffler, C. Hofmann, S. Kuhn, S. Reitzenstein, L. V. Keldysh, V. D. Kulakovskii, T. L. Reinecke, A. Forchel, Strong coupling in a single quantum dot–semiconductor microcavity system. *Nature* **432**, 197–200 (2004).

13. T. Yoshie, A. Scherer, J. Hendrickson, G. Khitrova, H. M. Gibbs, G. Rupper, C. Ell, O. B. Shchekin, D. G. Deppe, Vacuum Rabi splitting with a single quantum dot in a photonic crystal nanocavity. *Nature* **432**, 200–203 (2004).

14. A. Javadi, I. Söllner, M. Arcari, S. L. Hansen, L. Midolo, S. Mahmoodian, G. Kiršanskė, T. Pregnolato, E. H. Lee, J. D. Song, S. Stobbe, P. Lodahl, Single-photon non- linear optics with a quantum dot in a waveguide. *Nat. Commun.* **6**, 8655 (2015).

15. I. Söllner, S. Mahmoodian, S. L. Hansen, L. Midolo, A. Javadi, G. Kiršanskė, T. Pregnolato, H. El-Ella, E. H. Lee, J. D. Song, S. Stobbe, P. Lodahl, Deterministic photonemitter coupling in chiral photonic circuits. *Nat. Nanotechnol.* **10**, 775–778 (2015).

16. E. Waks, J. Vuckovic, Dipole induced transparency in drop-filter cavity-waveguide systems. *Phys. Rev. Lett.* **96**, 153601 (2006).

17. C. L. Cortes, Z. Jacob, Super-Coulombic atom–atom interactions in hyperbolic media. *Nat. Commun.* **8**, 14144 (2017).

18. A. Gonzalez-Tudela, D. Martin-Cano, E. Moreno, L. Martin-Moreno, C. Tejedor, F. J. Garcia-Vidal, Entanglement of two qubits mediated by one-dimensional plasmonic waveguides. *Phys. Rev. Lett.* **106**, 020501 (2011).

19. A. González-Tudela, J. I. Cirac, Exotic quantum dynamics and purely long-range coherent interactions in Dirac conelike baths. *Phys. Rev. A* **97**, 043831 (2018).

20. L. Ying, M. Zhou, M. Mattei, B. Liu, P. Campagnola, R. H. Goldsmith, Z. Yu, Extended range of dipole-dipole interactions in periodically structured photonic media. *Phys. Rev. Lett.* **123**, 173901 (2019).

21. I. García-Elcano, A. González-Tudela, J. Bravo-Abad, Tunable and robust long-range coherent interactions between quantum emitters mediated by weyl bound states. *Phys. Rev. Lett.* **125**, 163602 (2020).

22. R. Fleury, A. Alù, Enhanced superradiance in epsilon-near-zero plasmonic channels. *Phys. Rev. B* **87**, 201101 (2013).

23. H. Tang, X. Ni, F. Du, V. Srikrishna, E. Mazur, On-chip light trapping in bilayer moiré photonic crystal slabs. *Appl. Phys. Lett.* **121**, 231702 (2022).

24. K. J. Vahala, Optical microcavities. *Nature* **424**, 839–846 (2003).

25. A. Badolato, K. Hennessy, M. Atatüre, J. Dreiser, E. Hu, P. M. Petroff, A. Imamoglu, Deterministic coupling of single quantum dots to single nanocavity modes. *Science* **308**, 1158–1161 (2005).

26. Y. Cao, V. Fatemi, A. Demir, S. Fang, S. L. Tomarken, J. Y. Luo, J. D. Sanchez-Yamagishi, K. Watanabe, T. Taniguchi, E. Kaxiras, R. C. Ashoori, P. Jarillo-Herrero, Correlated insulator behaviour at half-filling in magic-angle graphene superlattices. *Nature* **556**, 80–84 (2018).

27. K. F. Mak, J. Shan, Semiconductor moiré materials. *Nat. Nanotechnol.* **17**, 686–695 (2022).

28. E. Y. Andrei, D. K. Efetov, P. Jarillo-Herrero, A. H. MacDonald, K. F. Mak, T. Senthil, E. Tutuc, A. Yazdani, A. F. Young, The marvels of moiré materials. *Nat. Rev. Mater.* **6**, 201–206 (2021).

29. X.-R. Mao, Z.-K. Shao, H.-Y. Luan, S.-L. Wang, R.-M. Ma, Magic-angle lasers in nanostructured moiré superlattice. *Nat. Nanotechnol.* **16**, 1099–1105 (2021).

30. D. X. Nguyen, X. Letartre, E. Drouard, P. Viktorovitch, H. C. Nguyen, H. S. Nguyen, Magic configurations in moiré superlattice of bilayer photonic crystals: Almost-perfect flatbands and unconventional localization. *Phys. Rev. Res.* **4**, L032031 (2022).

31. X. Wang, Z. Liu, B. Chen, G. Qiu, D. Wei, J. Liu, Experimental demonstration of high-efficiency harmonic generation in photonic moiré superlattice microcavities. *Nano Lett.* **24**, 11327–11333 (2024).

32. M. Oudich, X. Kong, T. Zhang, C. Qiu, Y. Jing, Engineered moiré photonic and phononic superlattices. *Nat. Mater.* **23**, 1169–1178 (2024).

33. P. Wang, Y. Zheng, X. Chen, C. Huang, Y. V. Kartashov, L. Torner, V. V. Konotop, F. Ye, Localization and delocalization of light in photonic moiré lattices. *Nature* **577**, 42–46 (2020).

34. D. Yu, G. Li, L. Wang, D. Leykam, L. Yuan, X. Chen, Moiré lattice in one-dimensional synthetic frequency dimension. *Phys. Rev. Lett.* **130**, 143801 (2023).

35. A. Raun, H. Tang, X. Ni, E. Mazur, E. L. Hu, GaN magic angle laser in a merged moiré photonic crystal. *ACS Photonics* **10**, 3001–3007 (2023).

36. J. Bao, Z. Fu, T. Pramanik, J. Mao, Y. Chi, Y. Cao, C. Zhai, Y. Mao, T. Dai, X. Chen, X. Jia, L. Zhao, Y. Zheng, B. Tang, Z. Li, J. Luo, W. Wang, Y. Yang, Y. Peng, D. Liu, D. Dai, Q. He, A. L. Muthali, L. K. Oxenløwe, C. Vigliar, S. Paesani, H. Hou, R. Santagati, J. W. Silverstone, A. Laing, M. G. Thompson, J. L. O'Brien, Y. Ding, Q. Gong, J. Wang, Very-large-scale integrated quantum graph photonics. *Nat. Photonics* **17**, 573–581 (2023).

37. J. M. Arrazola, V. Bergholm, K. Brádler, T. R. Bromley, M. J. Collins, I. Dhand, A. Fumagalli, T. Gerrits, A. Goussev, L. G. Helt, J. Hundal, T. Isacsson, R. B. Israel, J. Izaac, S. Jahangiri, R. Janik, N. Killoran, S. P. Kumar, J. Lavoie, A. E. Lita, D. H. Mahler, M. Menotti, B. Morrison, S. W. Nam, L. Neuhaus, H. Y. Qi, N. Quesada, A. Repingon, K. K. Sabapathy, M. Schuld, D. Su, J. Swinarton, A. Száva, K. Tan, P. Tan, V. D. Vaidya, Z. Vernon, Z. Zabaneh, Y. Zhang, Quantum circuits with many photons on a programmable nanophotonic chip. *Nature* **591**, 54–60 (2021).

38. C. Shang, K. Feng, E. T. Hughes, A. Clark, M. Debnath, R. Koscica, G. Leake, J. Herman, D. Harame, P. Ludewig, Y. Wan, J. E. Bowers, Electrically pumped quantum-dot lasers grown on 300 mm patterned Si photonic wafers. *Light Sci. Appl.* **11**, 299 (2022).

39. W.-Q. Wei, A. He, B. Yang, Z.-H. Wang, J.-Z. Huang, D. Han, M. Ming, X. Guo, Y. Su, J.-J. Zhang, T. Wang, Monolithic integration of embedded III-V lasers on SOI. *Light Sci. Appl.* **12**, 84 (2023).

40. E. Di Benedetto, A. Gonzalez-Tudela, F. Ciccarello, Dipole-dipole interactions mediated by a photonic flat band. arXiv:2405.20382 [quant-ph] (2024).

41. C.-Y. Hao, Z. Zhan, P. A. Pantaleón, J.-Q. He, Y.-X. Zhao, K. Watanabe, T. Taniguchi, F. Guinea, L. He, Robust flat bands in twisted trilayer graphene moiré quasicrystals. *Nat. Commun.* **15**, 8437 (2024).

42. Y. Wan, S. Zhang, J. C. Norman, M. J. Kennedy, W. He, S. Liu, C. Xiang, C. Shang, J.-J. He, A. C. Gossard, J. E. Bowers, Tunable quantum dot lasers grown directly on silicon. *Optica* **6**, 1394 (2019).

43. T. Zhou, M. Tang, G. Xiang, B. Xiang, S. Hark, M. Martin, T. Baron, S. Pan, J.-S. Park, Z. Liu, S. Chen, Z. Zhang, H. Liu, Continuous-wave quantum dot photonic crystal lasers grown on on-axis Si (001). *Nat. Commun.* **11**, 977 (2020).

44. C. Saadi, H. S. Nguyen, S. Cueff, L. Ferrier, X. Letartre, S. Callard, How many supercells are required for unconventional light confinement in moiré photonic lattices? *Optica* **11**, 245 (2024).

45. M. Fujita, S. Takahashi, Y. Tanaka, T. Asano, S. Noda, Simultaneous inhibition and redistribution of spontaneous light emission in photonic crystals. *Science* **308**, 1296–1298 (2005).

46. R. H. Brown, R. Q. Twiss, Correlation between photons in two coherent beams of light. *Nature* **177**, 27–29 (1956).

47. M. Bayer, G. Ortner, O. Stern, A. Kuther, A. A. Gorbunov, A. Forchel, P. Hawrylak, S. Fafard, K. Hinzer, T. L. Reinecke, S. N. Walck, J. P. Reithmaier, F. Klopf, F. Schäfer, Fine structure of neutral and charged excitons in self-assembled In(Ga)As/(Al)GaAs quantum dots. *Phys. Rev. B* **65**, 195315 (2002).

48. Z. Hao, K. Zou, Y. Meng, J.-Y. Yan, F. Li, Y. Huo, C.-Y. Jin, F. Liu, T. Descamps, A. Iovan, V. Zwiller, X. Hu, High-performance eight-channel system with fractal superconducting nanowire single-photon detectors. *Chip* **3**, 100087 (2024).

49. J. H. Quilter, A. J. Brash, F. Liu, M. Glässl, A. M. Barth, V. M. Axt, A. J. Ramsay, M. S. Skolnick, A. M. Fox, Phonon-assisted population inversion of a single InGaAs / GaAs quantum dot by pulsed laser excitation. *Phys. Rev. Lett.* **114**, 137401 (2015).

50. N. Coste, D. A. Fioretto, N. Belabas, S. C. Wein, P. Hilaire, R. Frantzeskakis, M. Gundin, B. Goes, N. Somaschi, M. Morassi, A. Lemaître, I. Sagnes, A. Harouri, S. E. Economou, A. Auffeves, O. Krebs, L. Lanco, P. Senellart, High-rate entanglement between a semiconductor spin and indistinguishable photons. *Nat. Photonics* **17**, 582–587 (2023).

51. M. Bayer, T. L. Reinecke, F. Weidner, A. Larionov, A. McDonald, A. Forchel, Inhibition and enhancement of the spontaneous emission of quantum dots in structured microresonators. *Phys. Rev. Lett.* **86**, 3168–3171 (2001).

52. D. Englund, D. Fattal, E. Waks, G. Solomon, B. Zhang, T. Nakaoka, Y. Arakawa, Y. Yamamoto, J. Vučković, Controlling the spontaneous emission rate of single quantum dots in a two-dimensional photonic crystal. *Phys. Rev. Lett.* **95**, 013904 (2005).

53. R. G. Hulet, E. S. Hilfer, D. Kleppner, Inhibited spontaneous emission by a Rydberg atom. *Phys. Rev. Lett.* **55**, 2137–2140 (1985).

54. P. Lodahl, A. Floris Van Driel, I. S. Nikolaev, A. Irman, K. Overgaag, D. Vanmaekelbergh, W. L. Vos, Controlling the dynamics of spontaneous emission from quantum dots by photonic crystals. *Nature* **430**, 654–657 (2004).

55. F. Liu, A. J. Brash, J. O'Hara, L. M. P. P. Martins, C. L. Phillips, R. J. Coles, B. Royall, E. Clarke, C. Bentham, N. Prtljaga, I. E. Itskevich, L. R. Wilson, M. S. Skolnick, A. M. Fox, High Purcell factor generation of indistinguishable on-chip single photons. *Nat. Nanotechnol.* **13**, 835–840 (2018).

56. T. H. Talukdar, A. L. Hardison, J. D. Ryckman, Moiré effects in silicon photonic nanowires. *ACS Photonics* **9**, 1286–1294 (2022).

57. D. X. Nguyen, X. Letartre, E. Drouard, P. Viktorovitch, H. C. Nguyen, H. S. Nguyen, Magic configurations in moiré superlattice of bilayer photonic crystal: Almost-perfect flatbands and unconventional localization. *Phys. Rev. Res.* **4**, L032031 (2022).

58. R.-M. Ma, H.-Y. Luan, Z.-W. Zhao, W.-Z. Mao, S.-L. Wang, Y.-H. Ouyang, Z.-K. Shao, Twisted lattice nanocavity with theoretical quality factor exceeding 200 billion. *Fundam. Res.* **3**, 537–543 (2023).

59. X. Gao, C. W. Hsu, B. Zhen, X. Lin, J. D. Joannopoulos, M. Soljačić, H. Chen, Formation mechanism of guided resonances and bound states in the continuum in photonic crystal slabs. *Sci. Rep.* **6**, 31908 (2016).

60. C. W. Hsu, B. Zhen, A. D. Stone, J. D. Joannopoulos, M. Soljačić, Bound states in the continuum. *Nat. Rev. Mater.* **1**, 16048 (2016).

61. D. C. Marinica, A. G. Borisov, S. V. Shabanov, Bound states in the continuum in photonics. *Phys. Rev. Lett.* **100**, 183902 (2008).

62. K. Koshelev, S. Lepeshov, M. Liu, A. Bogdanov, Y. Kivshar, Asymmetric metasurfaces with high-q resonances governed by bound states in the continuum. *Phys. Rev. Lett.* **121**, 193903 (2018).

63. A. Sipahigil, R. E. Evans, D. D. Sukachev, M. J. Burek, J. Borregaard, M. K. Bhaskar, C. T. Nguyen, J. L. Pacheco, H. A. Atikian, C. Meuwly, R. M. Camacho, F. Jelezko, E. Bielejec, H. Park, M. Lončar, M. D. Lukin, An integrated diamond nanophotonics platform for quantum-optical networks. *Science* **354**, 847–850 (2016).

64. Y.-M. He, G. Clark, J. R. Schaibley, Y. He, M.-C. Chen, Y.-J. Wei, X. Ding, Q. Zhang, W. Yao, X. Xu, C.-Y. Lu, J.-W. Pan, Single quantum emitters in monolayer semiconductors. *Nat. Nanotechnol.* **10**, 497–502 (2015).

65. A. E. K. Kaplan, C. J. Krajewska, A. H. Proppe, W. Sun, T. Sverko, D. B. Berkinsky, H. Utzat, M. G. Bawendi, Hong–Ou–Mandel interference in colloidal $CsPbBr_3$ perovskite nanocrystals. *Nat. Photonics* **17**, 775–780 (2023).

66. P. Michler, A. Kiraz, C. Becher, W. V. Schoenfeld, P. M. Petroff, L. Zhang, E. Hu, A. Imamoglu, A quantum dot single-photon turnstile device. *Science* **290**, 2282–2285 (2000).

67. X. Ding, Y.-P. Guo, M.-C. Xu, R.-Z. Liu, G.-Y. Zou, J.-Y. Zhao, Z.-X. Ge, Q.-H. Zhang, H.-L. Liu, L.-J. Wang, M.-C. Chen, H. Wang, Y.-M. He, Y.-H. Huo, C.-Y. Lu, J.-W. Pan, High-efficiency single-photon source above the loss-tolerant threshold for efficient linear optical quantum computing. arXiv:2311.08347 [quant-ph] (2023).

68. J. Liu, R. Su, Y. Wei, B. Yao, S. F. C. da Silva, Y. Yu, J. Iles-Smith, K. Srinivasan, A. Rastelli, J. Li, X. Wang, A solid-state source of strongly entangled photon pairs with high brightness and indistinguishability. *Nat. Nanotechnol.* **14**, 586–593 (2019).

69. M. B. Rota, T. M. Krieger, Q. Buchinger, M. Beccaceci, J. Neuwirth, H. Huet, N. Horová, G. Lovicu, G. Ronco, S. F. Covre da Silva, G. Pettinari, M. Moczała-Dusanowska, C. Kohlberger, S. Manna, S. Stroj, J. Freund, X. Yuan, C. Schneider, M. Ježek, S. Höfling, F. Basso Basset, T. Huber-Loyola, A. Rastelli, R. Trotta, A source of entangled photons based on a cavity-enhanced and strain-tuned GaAs quantum dot. *eLight* **4**, 13 (2024).

70. H. Siampour, C. ORourke, A. J. Brash, M. N. Makhonin, R. Dost, D. J. Hallett, E. Clarke, P. K. Patil, M. S. Skolnick, A. M. Fox, Observation of large spontaneous emission rate enhancement of quantum dots in a broken-symmetry slow-light waveguide. *NPJ Quantum Inf.* **9**, 15 (2023).

71. B. Hacker, S. Welte, G. Rempe, S. Ritter, A photon-photon quantum gate based on a single atom in an optical resonator. *Nature* **536**, 193–196 (2016).

72. H. Kim, R. Bose, T. C. Shen, G. S. Solomon, E. Waks, A quantum logic gate between a solid-state quantum bit and a photon. *Nat. Photonics* **7**, 373–377 (2013).

73. D. Niemietz, P. Farrera, S. Langenfeld, G. Rempe, Nondestructive detection of photonic qubits. *Nature* **591**, 570–574 (2021).

74. P. Thomas, L. Ruscio, O. Morin, G. Rempe, Efficient generation of entangled multiphoton graph states from a single atom. *Nature* **608**, 677–681 (2022).

75. S. Sun, H. Kim, Z. Luo, G. S. Solomon, E. Waks, A single-photon switch and transistor enabled by a solid-state quantum memory. *Science* **361**, 57–60 (2018).

76. H. J. Kimble, The quantum internet. *Nature* **453**, 1023–1030 (2008).

77. K. Hennessy, A. Badolato, M. Winger, D. Gerace, M. Atatüre, S. Gulde, S. Fält, E. L. Hu, A. Imamoğlu, Quantum nature of a strongly coupled single quantum dot–cavity system. *Nature* **445**, 896–899 (2007).

78. K. Kuruma, Y. Ota, M. Kakuda, S. Iwamoto, Y. Arakawa, Surface-passivated high-Q GaAs photonic crystal nanocavity with quantum dots. *APL Photonics* **5**, 046106 (2020).

79. R. Bose, D. Sridharan, H. Kim, G. S. Solomon, E. Waks, Low-photon-number optical switching with a single quantum dot coupled to a photonic crystal cavity. *Phys. Rev. Lett.* **108**, 227402 (2012).

80. T. Volz, A. Reinhard, M. Winger, A. Badolato, K. J. Hennessy, E. L. Hu, A. Imamoğlu, Ultrafast all-optical switching by single photons. *Nat. Photonics* **6**, 605–609 (2012).

81. T. Berstermann, T. Auer, H. Kurtze, M. Schwab, D. R. Yakovlev, M. Bayer, J. Wiersig, C. Gies, F. Jahnke, D. Reuter, A. D. Wieck, Systematic study of carrier correlations in the electron-hole recombination dynamics of quantum dots. *Phys. Rev. B* **76**, 165318 (2007).

82. G. Reithmaier, F. Flassig, P. Hasch, S. Lichtmannecker, K. Müller, J. Vučković, R. Gross, M. Kaniber, J. J. Finley, A carrier relaxation bottleneck probed in single InGaAs quantum dots

using integrated superconducting single photon detectors. *Appl. Phys. Lett.* **105**, 081107 (2014).

83. J.-Y. Yan, C. Chen, X.-D. Zhang, Y.-T. Wang, H.-G. Babin, A. D. Wieck, A. Ludwig, Y. Meng, X. Hu, H. Duan, W. Chen, W. Fang, M. Cygorek, X. Lin, D.-W. Wang, C.-Y. Jin, F. Liu, Coherent control of a high-orbital hole in a semiconductor quantum dot. *Nat. Nanotechnol.* **18**, 1139–1146 (2023).

84. E. A. Zibik, T. Grange, B. A. Carpenter, N. E. Porter, R. Ferreira, G. Bastard, D. Stehr, S. Winnerl, M. Helm, H. Y. Liu, M. S. Skolnick, L. R. Wilson, Long lifetimes of quantum-dot intersublevel transitions in the terahertz range. *Nat. Mater.* **8**, 803–807 (2009).
